# Sir David Barry

**Published:** 1836-04

**Authors:** 


### OBITUARY.

### SIR DAVID BARRY, M.D.

In our last number we announced the sudden and premature death of this amiable and estimable physician: we now add a few particulars of his life, omitted for want of room.

Sir David Barry was born on the 12th March, 1780, in the county of Ros common, in Ireland. We believe his parents were persons of great respectability, but in no elevated station in life. They, however, gave their son the most valuable of endowments, next to that of a sound physical constitution, a good education; and he was early distinguished both for his classical and mathematical attainments. Originally destined for another profession, at the commencement of the French war he turned his attention to medicine, and prepared himself for the public service, at that time the ready recipient of the aspiring and active youth of the country. Barry entered the medical department of the army as assistant-surgeon of the 87th regiment, on the 6th March, 1806. After three years' service in this capacity, he resigned his medical appointment, and took an ensign's commission in the same regiment, then serving in Portugal. He, however, did not long continue in his more warlike position, but returned to the medical service of the army, being appointed assistant-surgeon of the 58th Foot, on the 1st Feb. 1810. In this situation he had the good fortune to give important surgical aid to field-marshal Beresford, when wounded in the battle of Salamanca, and who ever after warmly espoused his interests. He was appointed surgeon to the Portuguese forces on the 25th March, 1813, and staff-surgeon in the British army on the 25th September, 1814. At the close of the war, Mr. Barry was nominated staff-surgeon of the district of Braganza, in Portugal, and resided for some years in this capacity at Oporto, where he married Miss Whately, sister of the present archbishop of Dublin. On the breaking out of the revolution in Portugal, in 1820, he returned to England; and, shortly afterwards, received the degree of M.D. from one of the Scottish universities, and became an extra licentiate of the London College of Physicians.

Dr. Barry proceeded to Paris in 1822, and devoted himself at once to the study and practice of his profession. In this capital he remained four years; and having qualified himself by the necessary attendance at the schools, took the degree of M.D. at the university, in 1827. It was in Paris that Dr. Barry made his various original and highly important experiments on the circulation of the blood in the veins, and the bearing of this on the function of absorption, and on the treatment of poisoning by external wounds. The result of these enquiries he submitted in the first instance to the Royal Institute of France, and to the Royal Academy of Medicine in Paris; and then published them in a separate form in London, in 1826, under the title of "Experimental Researches on the Influence exercised by Atmospheric Pressure upon the Progression of the Blood in the Veins, upon the function called Absorption, and upon the Prevention and Cure of the Symptoms caused by the Bites of Rabid or Venomous Animals." The great importance of this work was immediately recognized by the members of the profession, both in France and England; and, although some of the author's positions have been controverted, and some disproved, it will always remain a valuable addition to physiological science.

In 1826 Dr. Barry returned once more to England, and commenced practice in London; but his reputation and his connexion with the army did not permit him to remain long stationary. In 1828 he was sent to Gibraltar, in an official capacity, and with an especial view towards investigating the nature of the yellow fever, then prevalent in that garrison. He was promoted to the rank of physician to the forces on the 5th Nov. 1829; and returned with this rank to London, in 1830. Dr. Barry published various documents in the journals, particularly in the Medical and Physical Journal, on the subject of the fever; and, among others, a Letter to Sir James M'Grigor, "On the Sanitary Management of the Gibraltar Fever," which contains many valuable hygienic regulations for the prevention and suppression of this fatal epidemic. In the following year, in June, he was sent, in conjunction with Dr. William Russel, to St. Petersburg, with the view of investigating the nature of the cholera, then raging there, and spreading alarm through every other country. On his return from this mission, he was made deputy inspector general of hospitals, on the 16th Dec. of the same year. When the cholera appeared in England, Dr. Barry was much employed in its investigation by government; and, in conjunction with Dr. now Sir William Russel, constituted, for a time, the medical part of the official board for its supervision. In this capacity, as also in that of commissioner to Russia, he published various memoirs on the nature and mode of preventing the cholera, which were very creditable to his penetration and industry, and proved useful at the time. For his services in this department he received the honour of knighthood from the king, having been previously invested with the dignities of knight of the order of the Tower and Sword of Portugal, and of St. Anne of Russia.

In the year 1833, Sir David Barry was appointed one of the commissioners for enquiring into the health of children employed in the British factories; and in 1834 he was nominated to the Irish commission for investigating the state of the poor, and of the medical charities in Ireland. He had only recently returned from this last enquiry, and was still busily occupied in collecting and preparing his voluminous and valuable documents on this most important department of statistics, when he was suddenly cut off in the flower of his useful and active life, on the 4th of November, 1836. The cause of his death was the rupture of an aneurism of the aorta, with which he must have been long affected, although its presence had scarcely been indicated by symptoms, and certainly was never suspected either by himself or any of his medical friends.

Sir David Barry was an honorable and honored member of the profession. He was a man of much information, both general and professional; an extremely agreeable companion, and highly popular among his brethren.

---

